# A surface plasmon resonance based approach for measuring response to pneumococcal vaccine

**DOI:** 10.1038/s41598-021-85958-0

**Published:** 2021-03-22

**Authors:** Marta Garrido-Jareño, Leonor Puchades-Carrasco, Leticia Orti-Pérez, José Miguel Sahuquillo-Arce, María del Carmen Meyer-García, Joan Mollar-Maseres, Carmina Lloret-Sos, Ana Gil-Brusola, José Luis López-Hontangas, José Manuel Beltrán-Garrido, Javier Pemán-García, Antonio Pineda-Lucena

**Affiliations:** 1 Drug Discovery Unit, Health Research Institute La Fe, Valencia, Spain; 2 Microbiology Department, University and Polytechnic Hospital La Fe, Valencia, Spain; 3 Preventive Medicine Department, University and Polytechnic Hospital La Fe, Valencia, Spain; 4 Severe Infection Group, Health Research Institute Hospital La Fe, Valencia, Spain; 5 Molecular Therapeutics Program, Center for Applied Medical Research, University of Navarra, Pamplona, Spain

**Keywords:** Microbiology, Diseases, Health care, Medical research

## Abstract

Incidence of pneumococcal disease has increased worldwide in recent years. Response to pneumococcal vaccine is usually measured using the multiserotype enzyme-linked immunosorbent assay (ELISA) pneumococcal test. However, this approach presents several limitations. Therefore, the introduction of new and more robust analytical approaches able to provide information on the efficacy of the pneumococcal vaccine would be very beneficial for the clinical management of patients. Surface plasmon resonance (SPR) has been shown to offer a valuable understanding of vaccines’ properties over the last years. The aim of this study is to evaluate the reliability of SPR for the anti-pneumococcal capsular polysaccharides (anti-PnPs) IgGs quantification in vaccinated. Fast protein liquid chromatography (FPLC) was used for the isolation of total IgGs from serum samples of vaccinated patients. Binding-SPR assays were performed to study the interaction between anti-PnPs IgGs and PCV13. A robust correlation was found between serum levels of anti-PnPs IgGs, measured by ELISA, and the SPR signal. Moreover, it was possible to correctly classify patients into “non-responder”, “responder” and “high-responder” groups according to their specific SPR PCV13 response profiles. SPR technology provides a valuable tool for reliably characterize the interaction between anti-PnPs IgGs and PCV13 in a very short experimental time.

## Introduction

*Streptococcus pneumoniae* represents a public health hazard as it is the most common cause of bacterial pneumonia, especially in children, the elderly and immunosuppressed patients, and causes 1.6 million annual deaths worldwide^1^. The severity, global nature of these infections and the emergence of antibiotic resistance makes vaccination a valuable health resource for prevention^2^. However, the complexity of the data generated from serotype-specific assays, historical variations in the assessment of pneumococcal antibodies and cut-off points that define response difficult the interpretation of what constitutes an adequate response^3^. In addition, pneumococcal vaccines are commonly employed as a tool to functionally evaluate a patient's humoral immune response^4^; i.e., anti-pneumococcal antibodies (PnAb) are measured before and after vaccination in order to determine whether an appropriate response has occurred.

Different pneumococcal vaccines have been developed which differ in two fundamental characteristics: the number of serotypes represented in the vaccine and the antigenic nature of the pneumococcal materials used. Contrary to adults, for whom recent studies increasingly recommended routine vaccination, the childhood immunization schedule is well known and generally well implemented in developed countries^5^. Two pneumococcal vaccines are currently widely used worldwide: Pneumovax 23 (PPV23; Merck, West Point, Pa.), a 23-valent vaccine containing 23 pneumococcal capsular polysaccharides (PnPs) serotypes, and Prevnar 13 (PCV13; Wyeth, Philadelphia, PA.), a 13-valent conjugate vaccine containing 13 PnPs serotypes and a non-toxic variant of diphtheria toxin (diphtheria CRM197 protein) which transforms the T cell-independent polysaccharide vaccines to T cell-dependent antigenic vaccines that are much more immunogenic, allowing its use in children under two years^6^.

Many methods have been used for the measurement of serotype-specific PnAb levels, the most common being the multiserotype anti-PnPs IgGs ELISA (Enzyme-Linked ImmunoSorbent Assay). This test allows the quantification of anti-PnPs IgGs for various serotypes of *S. pneumoniae* in only one determination. Limitations of this approach include the multiple reagents used, the high rate of false positive/negative results and the long experimental time required (about 2 h)^7–11^.

Surface plasmon resonance (SPR) is an analytical technique that can provide a deeper characterization of the antibody-antigen interaction, very valuable for a better understanding of a patient’s vaccine response. This experimental tool has greatly contributed to the research, development, and production of new vaccines in recent years^12,13^. SPR is based on an optical phenomenon that occurs when polarized light is reflected by a surface coated with a fine layer of metal (e.g., gold or silver). The binding of biomolecules on the sensor surface results in a change in the refractive index, which can be measured as a change in resonance angle or resonance wavelength. Thus, one of the interacting molecules (ligand) is immobilized on the surface of a sensor chip while the other (analyte) is injected in solution, flowing over the sensor surface. The change in refractive index on the surface has a linear relationship with the number of molecules bound to the sensor surface^14^. Recent studies based in this technology have shown that SPR-based methods are sensitive enough to detect antigen-specific IgGs in the ng/uL range, providing information for differentiating the antibody responses of immunized patients^15,16^. In a more recent study, Khurana et al*.* demonstrated the impact of repeated vaccinations on antibody-affinity maturation using this analytical approach^17,18^. Based on these previous reports, the aim of this pilot study was to evaluate the potential of SPR for the quantification of anti-PnPs IgGs in serum of patients after PCV13 vaccination, with a focus on the feasibility of implementing this experimental approach in routine clinical practice.

## Results

### Immunization state

Demographic characteristics and classification of patients included in the study according to their vaccine response profile, based on their anti-PnPs IgGs levels (ELISA), are summarized in Table 1. No significant age differences were observed among groups (ANOVA test *p *value = 0.99).

**Table 1:** Demographic characteristics of subjects included in the groups of study, classified according to their PCV13 response profile as determined by ELISA.

Vaccine response profile	N total	Age (years) (mean ± SD)	Anti-PnPs IgGs^a^ (mg/L) (mean ± SD)
High responder	8	43.50 ± 14.25	352.42 ± 50.33
Responder	10	43.10 ± 10.05	143.99 ± 74.38
Non-responder	6	43.00 ± 14.57	25.71 ± 13.66

*SD* Standard deviation.

^a^Determined by ELISA.

### Isolation of total IgG from serum samples

**Figure 1:**
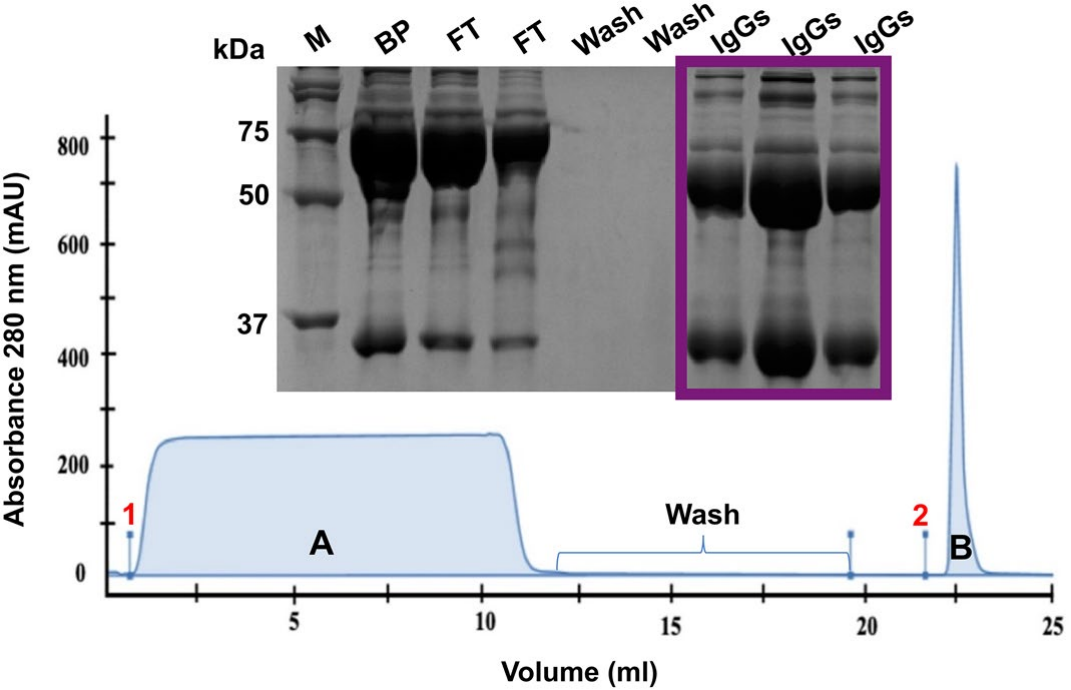
Total IgG isolation from serum samples following FPLC assay. Chromatogram corresponding to the interaction between serum proteins and HiTrap Protein G HP columns: (1) Diluted serum sample injection; (2) Glycine buffer injection. Peak A corresponds to the flow-through fractions, containing albumin and other serum proteins not retained in the column. Peak B (purple) corresponds to the elution fractions containing total IgGs present in serum samples. Peak composition was confirmed by SDS-PAGE 12% (top insert). M: Protein molecular weight marker; BP: Serum sample before purification, FT: flow through, containing albumin (66.5 KDa) and other serum proteins, IgGs: Elution fractions containing total IgGs present in serum samples (IgG’s light and heavy chains, 25 and 50 kDa, respectively).

Albumin and other high-concentrated serum proteins were efficiently removed from serum samples before SPR analysis to avoid potential interferences resulting from non-specific binding (NSB) to the chip. Thus, higher specificity in the interaction between specific anti-PnPs IgGs present in the serum and PCV13 immobilized on the chip could potentially be achieved. The composition of the different elution fractions obtained after the purification was analysed by SDS-PAGE (Fig. 1). A broad and flattened peak was obtained for the flow-through fractions, containing albumin and other serum proteins not retained in the column, while a narrow peak, corresponding to IgGs enriched elutions was obtained after the purification.

### Optimization of SPR binding assay conditions

**Figure 2:**
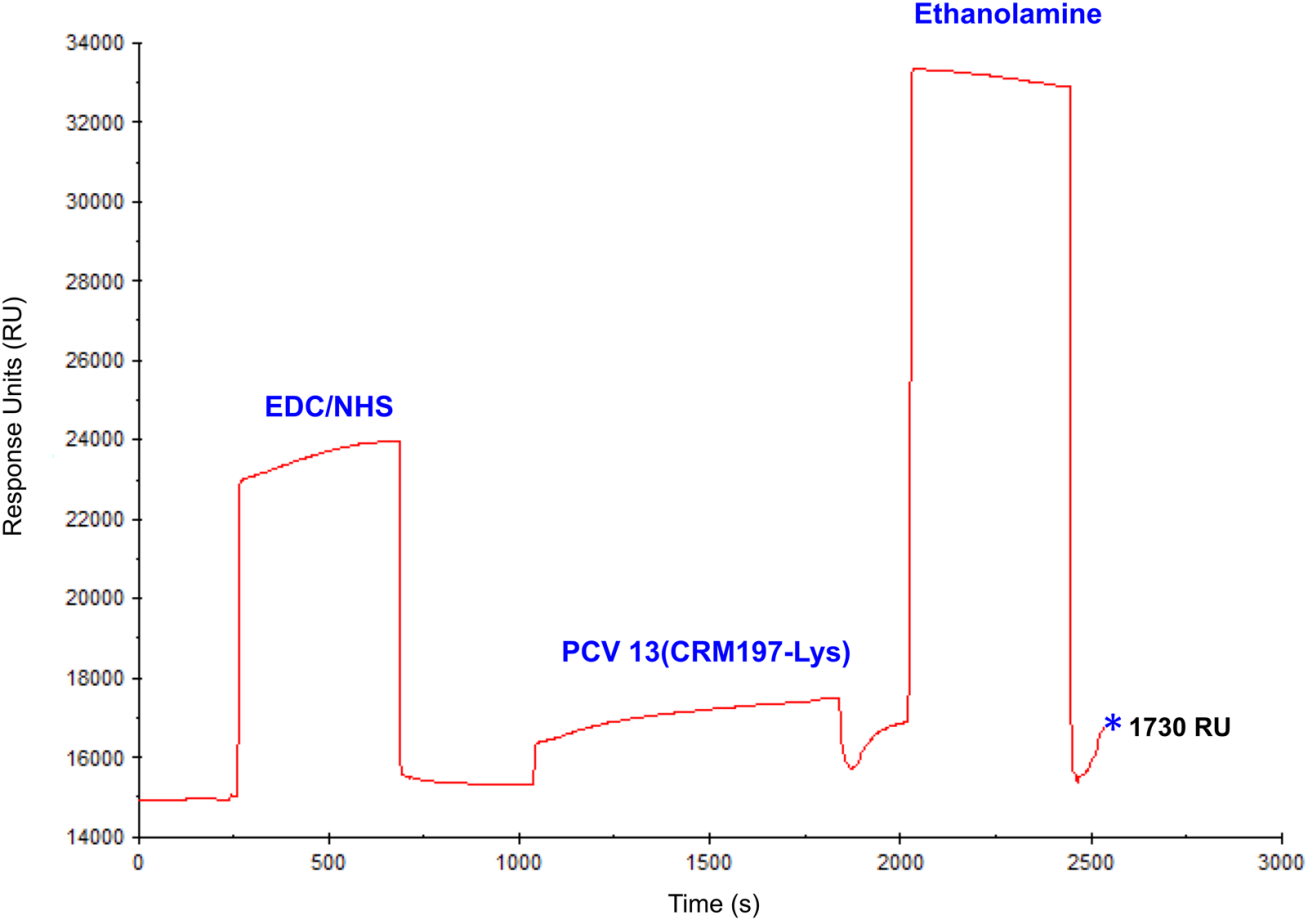
PCV13 immobilization using amine chemistry. Representative SPR sensorgram illustrating the steps of the immobilization process: (1) EDC/NHS were injected to activate the surface of the CM5 chip by modification of the carboxymethyl groups to N-Hydroxysuccinimide esters; (2) Diluted PCV13 was injected and immobilized in the chip surface by spontaneous reaction of the N-Hydroxysuccinimide esters in the chip surface with the amine groups of lysine residues present in PCV13′s diphtheria CRM197 protein; (3) Ethanolamine was injected to block free activated carboxymethyl groups in the chip surface, preventing further unspecific bindings during the SPR experiments. Blue asterisk indicates total immobilization level obtained in the chip (1730 RU).

**Figure 3:**
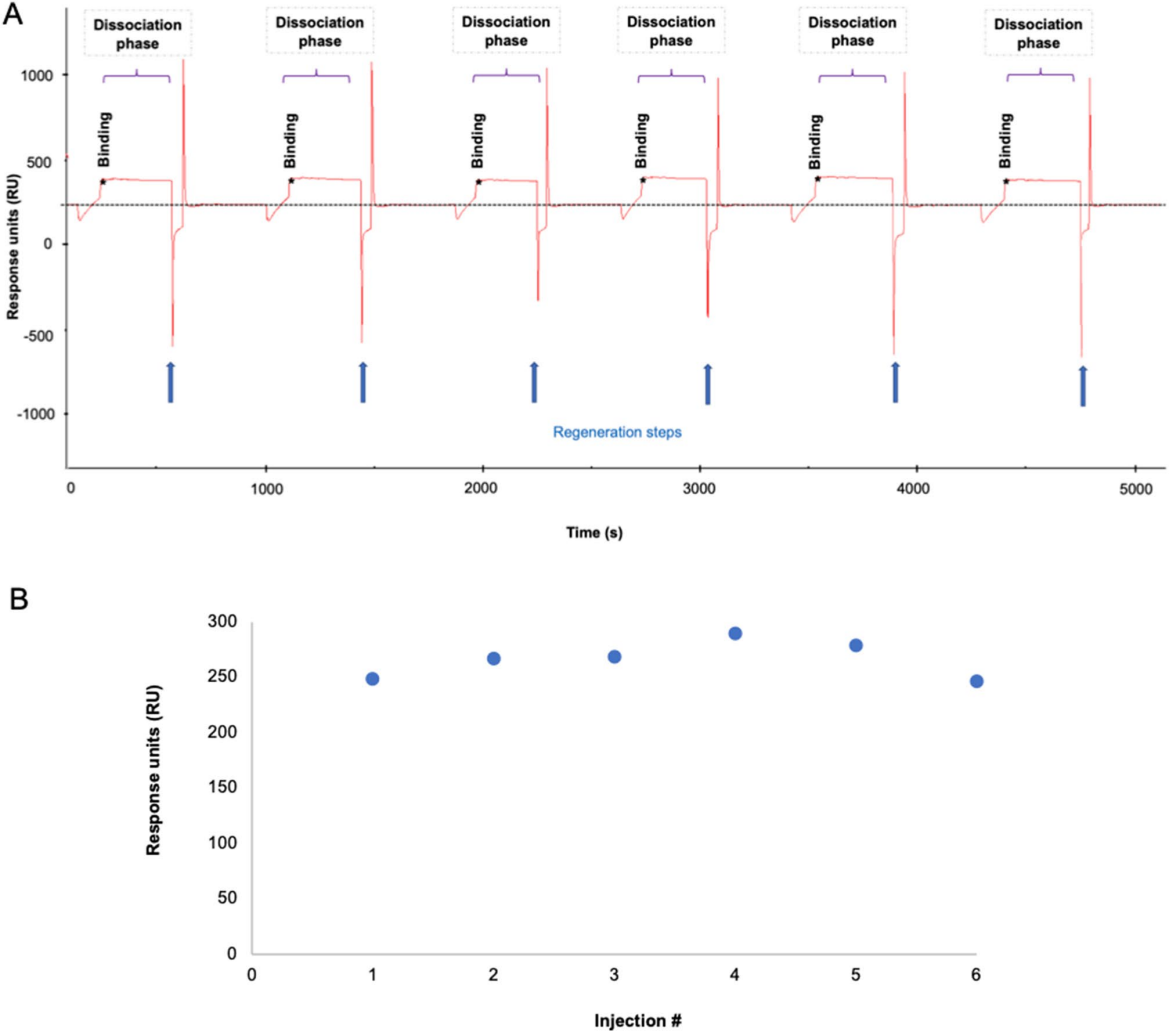
Efficacy of chip regeneration following repeated injections. (**A**) SPR sensorgrams of 6 consecutive injections for the same 1:8 diluted serum sample. Blue arrows indicate regeneration steps after each individual injection; (**B**) Graphical representation of SPR response obtained after each injection and its dependency with the injection number. * Binding report point refers to the maximum level of RU obtained after serum injection.

SPR binding experiments were performed after PCV13 immobilization at 1730 RUs, following chip activation (Fig. 2). The performance of different acid and basic solutions (i.e., glycine pH 2–3.5, HCl, NaCl and NaOH at different concentrations) was evaluated for the optimal regeneration of the chip surface (data not shown). An optimal regeneration of the chip surface was obtained after a 30 s injection of a 50 mM NaOH and 1 M NaCl solution. The same baseline level before and after injection and reproducible SPR response even after numerous cycles was achieved following this protocol (Fig. 3). After optimization of an effective regeneration protocol, different sample dilutions were assayed to ensure minimal NSB and best signal-to-noise ratio. Thus, the choice of the optimal dilution to perform the SPR experiments was based on the analysis of the reproducibility and the dynamic range of the SPR signal.

Intra-dilution reproducibility was assessed based on the mean of the coefficient of variation (%CV) obtained for all the samples included in the study. Thereby, non-diluted samples (1:1) showed the highest intra-dilution variability (%CV = 15.40%), while it was below 10% for all assayed dilutions. Particularly, dilutions 1:8 and 1:16 exhibited the highest reproducibility, with %CVs of 4.20 and 3.90, respectively (Table 2). Regarding the dynamic range of 1:8 and 1:16 dilutions, the highest fold change between the SPR signal for the most and the less anti-PnPs IgGs concentrated samples included in the study, as determined by ELISA (429.13 mg/L and 8.65 mg/L, respectively), were achieved for the 1:16 diluted samples, as is shown in Table 2. Overall, dilution 1:16 showed the SPR response with the lowest NSB and best signal-to-noise ratio for clear differentiation between different anti-PnPs IgGs concentrations^19,20^.

**Table 2:** Evaluation of different sample dilutions and effect on SPR parameters.

Sample dilution	Maximum SPR signal*^,a^	Minimum SPR signal*^,b^	FC*^,c^	%CV*^,d^
1:1	907.40	159.48	5.69	15.40
1:2	706.45	52.40	13.48	8.90
1:4	399.60	33.03	12.10	5.13
1:8	251.51	16.52	15.22	4.20
1:16	146.33	8.26	17.72	3.90
1:32	85.23	4.13	20.64	5.54

*Values in Response Units (RU).

^a^Corresponding to most anti-PnPs IgGs concentrated sample included in the study, as determined by ELISA (429.13 mg/L).

^b^Corresponding to less anti-PnPs IgGs concentrated sample included in the study, as determined by ELISA (8.65 mg/L).

^c^Fold Change between the RU values of the maximum and minimum SPR signal determined for each sample dilution.

^d^Mean of the coefficient of variation between replicates for all samples included in the study.

Based on these considerations, sample dilution 1:16 was selected as the best experimental condition, showing the most accurate and reproducible SPR results.

### Correlation between SPR response and anti-PnPs IgGs levels determined by ELISA

**Figure 4:**
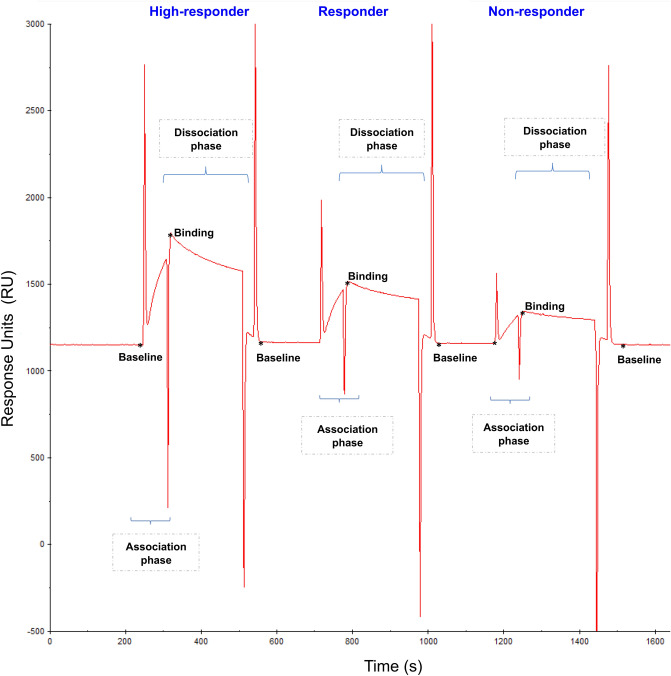
Representative SPR sensorgrams corresponding to the different groups of subjects included in the study. During SPR experiments, interaction between the PCV13 molecules immobilised in the chip and the serum anti-PnPs IgG occurs, changing the refractive index at the surface of the gold film and increasing signal intensity. Response units (RU) are used to describe the increase in the signal, where 1 RU is equal to a critical angle shift of 10^−4^ deg. At the start of the experiment all immobilised PCV13 molecules have not been exposed to serum anti-PnPs IgGs and the RU correspond to the starting critical angle (baseline). During the association phase, serum samples are injected into the flow cell and serum anti-PnPs IgGs bind to the immobilised PCV13 molecules, increasing the signal intensity. The shape of this curve can be used to measure the rate of association. This maximum RU relates to the maximum concentration of serum anti-PnPs IgGs that specifically interact with immobilized PCV13. During the dissociation phase, serum anti-PnPs IgGs are removed from the chip by the continuous flow. The surface is regenerated at the end of the experiment and returned to the starting critical angle (baseline) to start the next experiment. *Characteristically report points of SPR sensorgram: Baseline report point refers to the value of the critical angle at the star of the experiment. Binding report point refers to the maximum level of RU obtained after serum injection.

**Figure 5:**
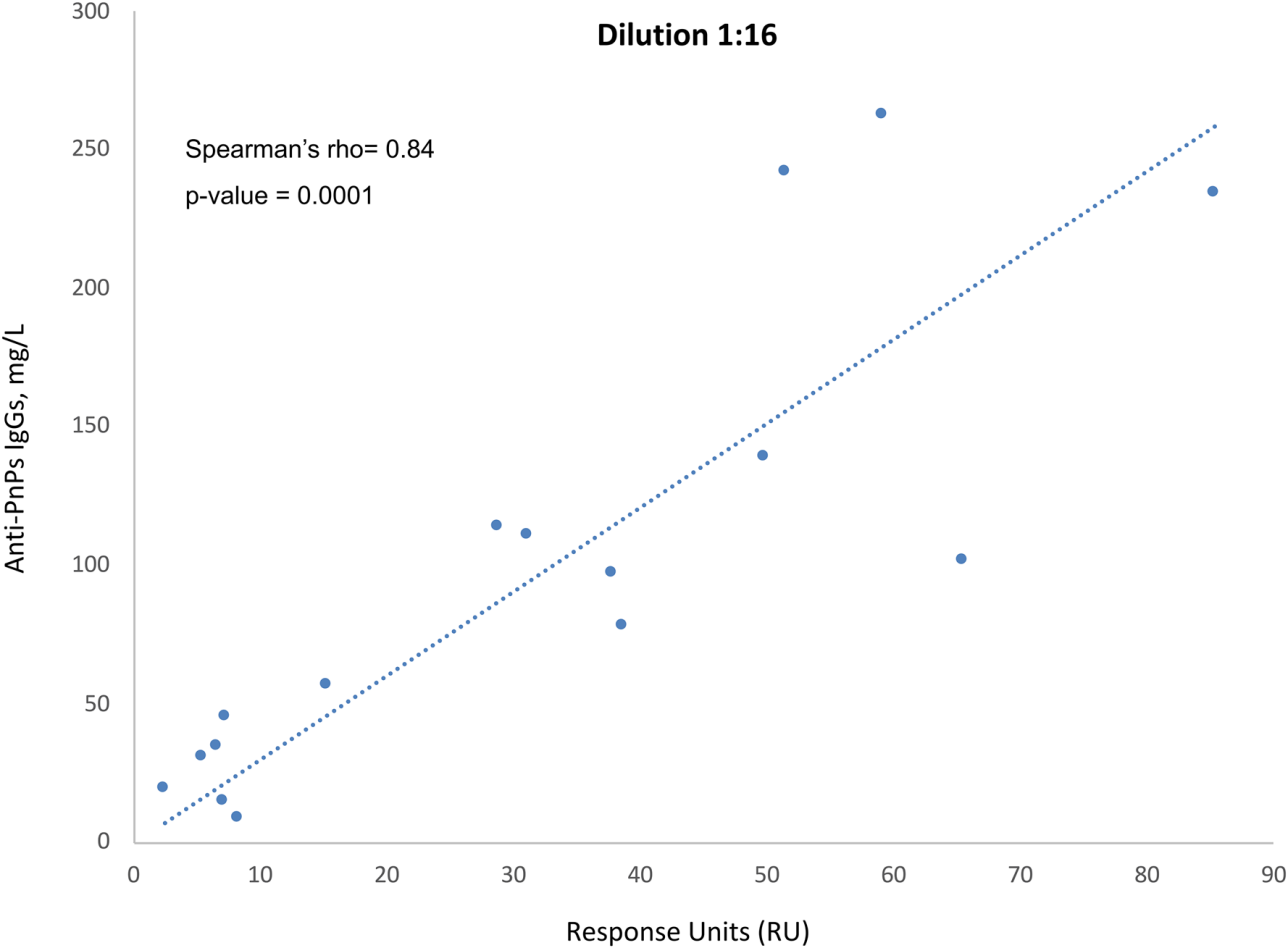
Correlation between SPR response and serum anti-PnPs IgGs levels determined by ELISA. Spearman’s rank correlation analysis between SPR response (RU) and serum anti-PnPs IgGs levels determined by ELISA (mg/L) for all samples included in the study with a serum anti-PnPs IgGs concentration below 270 mg/L, as determined by ELISA (SPR results for the 1:16 dilution).

**Figure 6:**
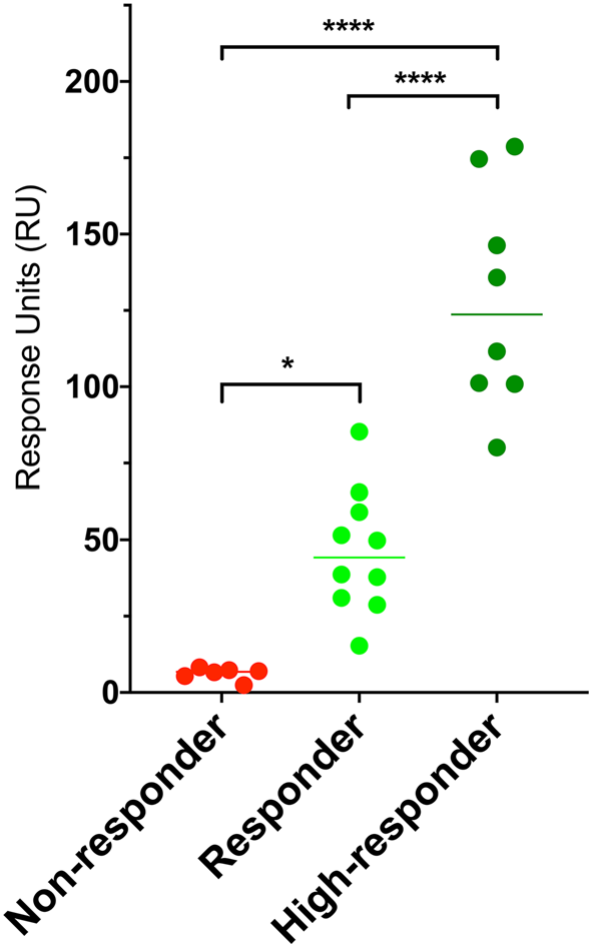
SPR binding profile reflects subjects’ immunization state. Dot plots showing the SPR binding level (1:16 dilution) in the samples included in the study, classified according to their PCV13 response profile, determined by ELISA: Non-responder (red), Responder (light green), High-responder (dark green). Student’s t-test: *p value < 0.05; ****p value < 0.0001.

SPR binding profiles of subjects included in the study reflected patients’ immunization state (Fig. 4). A robust correlation between the SPR response and the concentration of anti-PnPs IgGs determined by ELISA was found for all samples included in the study using the 1:16 dilution (Spearman’s rho = 0.84, *p *value = 0.001) (Fig. 5). Only samples with a serum anti-PnPs IgGs concentration below 270 mg/L, as determined by ELISA, were included in these analyses to avoid potential interferences due to the saturation of the ELISA signal. This strong correlation was also observed when analyzing all the different dilutions assayed during the study (Fig. S1). Moreover, the analysis of the linearity of the SPR signal within this dynamic range (Fig. S2) revealed a good linearity of the SPR response for the different sample dilutions analyzed in the study (R^2^ = 0.99). Finally, statistically significant differences in SPR response were found between the groups of patients classified according to anti-PnPs IgGs levels determined by ELISA (Fig. 6; *p* value < 0.0001).

## Discussion

Recent studies have described that SPR could become a relevant tool for the design and evaluation of vaccines for the clinical management of several infectious diseases, such as HIV and Hepatitis^16,21,22^. However, to the best of our knowledge, our study demonstrates for the first time that SPR-based approaches may provide highly reliable and accurate measurements of serum anti-PnPs IgGs in a very short experimental time (2 min). Moreover, SPR assays could offer additional advantages in routine clinical practice over classic ELISA. Thus, it would be possible to regenerate the sensor and run over two hundred analytical samples using the same chip, which would also translate into a better cost-effectiveness ratio over commercial ELISA kits.

In this study, different experimental actions were taken to ensure minimal NSB and optimal signal-to-noise ratios in SPR experiments^23–25^. First, a Protein G Sepharose High Performance FPLC method was used to purify serum total IgGs, as it as previously described^26–28^. Then, taking advantage of amine groups of lysine residues present in PCV13′s diphtheria CRM197 protein, amine coupling was chosen for PCV13 immobilization on a CM5 chip surface. Following this approach, a good PCV13 immobilization level was achieved. Moreover, this immobilization strategy facilitated the interaction between anti-PnPs IgGs present in the serum and the PCV13 immobilized in the chip, as no covalent bond is generated between PCV13 and the surface chip, ensuring accessibility to the active site^29^. Finally, in order to test the reusability and durability of the bioactive surface, different regeneration conditions were evaluated. An effective regeneration protocol should remove anti-PnPs IgGs bound to PCV13, while PCV13 molecules remain immobilized in the chip surface. In fact, as shown in Fig. 3, an excellent recovery of the sensorgram baseline was obtained using an optimized chip regeneration protocol^30^.

A robust correlation was also found when comparing the SPR response levels and the anti-PnPs IgGs concentration, as determined by ELISA, for all patients included in the study. A good sensitivity and optimal reproducibility levels were obtained when 1:16 diluted serum samples were used for the SPR experiments. The optimization of the assay conditions allowed the performance of SPR experiments without the need to saturate the ligand in order to get meaningful results. This result constitutes a relevant finding because higher analyte concentrations can give rise to multiphasic binding curves^31,32^. Moreover, removal of non-specific IgGs from the the chip was obtained by the continuous flow of buffer, as it is achieved with the washes during the ELISA tests^31,32^. The 1:16 dilution of the samples provided the minimal variability in comparison with the other assayed sample dilutions. In particular, the highest variation between replicates, over 10%, was observed for samples diluted 1:1. A potential explanation for this finding could be that, at higher concentrations, IgGs could aggregate and bind to each other during the SPR binding experiment. This phenomenon would explain the differences found in the SPR signal when replicating this condition^31^. Overall, the 1:16 dilution was deemed optimal because it provided a wide dynamic range of SPR signals that could facilitate the discrimination of different anti-PnPs IgGs concentrations^20^.

In summary, the interaction between anti-PnPs IgGs and PCV13 with a high specificity was successfully measured using a SPR-based approach. Furthermore, significant differences in the SPR binding profile of patients were found for patients classified according to their PCV13 response profile, as determined by serum anti-PnPs IgGs levels measured by ELISA. Although further investigations in larger cohorts of patients and using different pneumococcal vaccines are still needed, the results from this pilot study clearly show that SPR is a very valuable tool for measuring response to pneumococcal vaccine in the clinical practice.

Finally, most current pneumococcal vaccination schemes are based on repeated doses of the same vaccine to ensure the production of protective levels of IgGs, although some reports propose that natural infection may induce stronger and longer-lasting immunity than vaccines^33,34^. Nowadays, several limitations difficult the interpretation of what constitutes an adequate vaccine response^3^. In future studies, SPR could represent a very promising tool for evaluating and measuring the effect of different vaccines and specific serotypes following different vaccination schemes, allowing to determine whether an appropriate response has been achieved.

## Methods

### Study design

A total of 24 patients were enrolled in this study at the Department of Preventive Medicine of the University and Polytechnic La Fe Hospital (Valencia, Spain). Serum samples were collected one month after vaccination with PCV13. Exclusion criteria included patients aged less than 18 years old, patients with previous pneumococcal infection, as well as those who had received a previous dose of PCV13 in the last 5 years. Recruitment and sampling procedures were performed in accordance with the Declaration of Helsinki, and after approval from the Ethics Committee of the Health Research Institute La Fe (IIS La Fe, Valencia, Spain). Written informed consent was obtained from each participant included in this study.

### Quantification of serum anti-PnPs antibodies by ELISA

VaccZyme Anti-PCP IgG Enzyme Immunoassay Kit (The Binding Site Group, Birmingham, UK) was used to assess anti-PnPs IgG titers, according to the manufacturer’s instructions. Patient’s classification was performed based on the measuring range of the ELISA assay (3.3–270 mg/L) and following the Binding Site Group recommendations into: (i) PCV13 non/poor-responders (anti-PnPs IgGs ≤ 50 mg/L), (ii) PCV13 responders (anti-PnPs IgGs = 51–270 mg/L), and (iii) PCV13 high-responders (anti-PnPs IgGs > 270 mg/L)^35,36^.

### Isolation of serum total IgGs

Total IgGs were isolated from serum using fast protein liquid chromatography (FPLC) in an ÄKTA pure chromatography system (GE Healthcare, Uppsala, Sweden). HiTrap HP columns, prepacked with Protein G Sepharose High Performance (GE Healthcare, Uppsala, Sweden), were used to isolate IgGs from serum samples. Briefly, sera were diluted (1:35, v/v, in 20 mM NaH_2_PO_4_ buffer, pH = 7), applied to the HiTrap Protein G HP column at 1 ml/min, and eluted using a glycine buffer (100 mM Glycine, pH = 2.7; 1 ml/min). IgG-containing elution fractions, as detected by SDS-PAGE, were dialyzed against HEPES buffer (10 mM HEPES, 150 mM NaCl, pH = 7.4) for 24 h at 4ºC, then concentrated using 10 KDa centrifugal filter units Amicon Ultra 10 K (Millipore, Bedford, USA) at 5.000 rpm up to a concentration of 5–7 mg/ml. A mass extinction coefficient (ε) of 13.7 M^-1^ cm^-1^ was used for measuring the IgG concentration in a NanoDrop One (Thermo Fisher, Madison, USA).

### Quantification of serum anti-PnPs antibodies by SPR

The binding between anti-PnPs IgGs and PCV13 was monitored at 25 °C using a Biacore X100 instrument (GE Healthcare).

#### PCV13 preparation

PCV13 was dehydrated using Microcon centrifugal filter unit YM-50 membrane Amicon (10.800 rpm, 4ºC) and recovered in 320 µl of 10 mM sodium acetate buffer (pH 4.5), to obtain a final concentration of 100 µg/ml diphtheria toxoid (CRM197).

#### PCV13 immobilization

Free amines of CRM197 (100 µg/ml) contained in PCV13 were covalently immobilized in the flow cell 2 of a carboxy-methylated CM5 sensor chip by (i) activating the carboxymethyl groups with 70µL of N-hydroxysuccinimide (NHS) and 1-ethyl-3-(3-dimethylaminopropyl)-carbodiimide hydrochloride (EDC) solution (10 µl/min), (ii) injecting 50 µl of PCV13 to promote the coupling reaction (10 µl/min), and (iii) blocking the free activated carboxymethyl groups with 70 µl of ethanolamine (10 µl/min) (Fig. S3). A reference channel (flow cell 1) was immobilised in parallel, without PCV13, following the same procedure. After immobilization, 10 injections of 5 µl of regeneration solution (50 mM NaOH and 1 M NaCl) were performed until baseline stabilization was obtained (10 µl/min).

#### Binding SPR assay

For the evaluation of binding, serial dilutions (1:1, 1:2, 1:4, 1:8, 1:16, 1:32) of purified IgG samples were freshly prepared in running buffer HBS-EP (10 mM HEPES, 500 mM NaCl, 3 mM EDTA, 0.1% surfactant P20, pH = 7.4), previously filtered and degassed. A total volume of 10µL were injected for each dilution (60 s contact time; 10 µl/min) for association. Dissociation was performed over a 200 s interval. Surface regeneration between samples was carried out injecting 5 µl of regeneration solution (50 mM NaOH and 1 M NaCl) during 30 s (10 µl/min). Triplicates of the samples were injected for all dilutions.

#### Data analysis

Experimental results were analysed using Biacore X100 Evaluation Software 2.0.1 (GE Healthcare, Uppsala, Sweden). The interaction between injected anti-PnPs IgGs and PCV13 immobilised in the CM5 sensor chip was defined by the binding response. The corrected response for flow cell 2, in response units (RU), was obtained by subtraction of the RU detected in the reference channel (flow cell 1) and was characterized using the evaluation methods predefined by GE Healthcare (GE Healthcare, Uppsala, Sweden).

### Statistical analysis

Results were analysed using the “ClickR” package (v0.4.3.2)^37^. Spearman’s rank correlation analyses were performed for the evaluation of the correlation between anti-PnPs IgGs concentrations (ELISA), and the binding response (SPR). ANOVA and Student’s t-test were used to assess the statistical significance of the differences in SPR binding results among the groups of patients. A *p* value < 0.05 was considered significant.

## Supplementary Information

Supplementary Information
