# Supplementary material for: A surface plasmon resonance based approach for measuring response to pneumococcal vaccine
Source: Sci Rep. 2021 Mar 22;11:6502. doi: 10.1038/s41598-021-85958-0 (PMC7985148; doi:10.1038/s41598-021-85958-0)
Supplement: Supplementary file 1 — Supplementary Information [file 41598_2021_85958_MOESM1_ESM.docx]

*Article*

**A Surface Plasmon Resonance based approach for measuring response to pneumococcal vaccine**

**Marta Garrido-Jareño^a,b,1^, Leonor Puchades-Carrasco^a,1^, Leticia Orti-Pérez^a^, José Miguel Sahuquillo-Arce^b^, María del Carmen Meyer-García^c^, Joan Mollar-Maseres^c^, Carmina Lloret-Sos^b^, Ana Gil-Brusola^b,d^, José Luis López-Hontangas^b^, José Manuel Beltrán-Garrido^c^, Javier Pemán-García^b,d,**^, and Antonio Pineda-Lucena^a,e,*^**

^a^Drug Discovery Unit, Health Research Institute La Fe (Valencia, Spain)

^b^Microbiology Department, University and Polytechnic Hospital La Fe (Valencia, Spain)

^c^Preventive Medicine Department, University and Polytechnic Hospital La Fe (Valencia, Spain)

^d^Severe Infection Group, Health Research Institute La Fe (Valencia, Spain)

^e^Molecular Therapeutics Program, Center for Applied Medical Research, University of Navarra (Pamplona, Spain)

^1^M Garrido-Jareño and L Puchades-Carrasco contributed equally to this article.

*** Corresponding author.**

Antonio Pineda-Lucena

Molecular Therapeutics Program, Center for Applied Medical Research, University of Navarra (Pamplona, Spain)

E-mail address: [apinedal@unav.es](mailto:apinedal@unav.es)

Telephone number: +34 948194700, #772044, #812044

**** Alternate corresponding author.**

Javier Pemán-García

Severe Infection Group, Health Research Institute Hospital La Fe (Valencia, Spain)

E-mail address: [peman_jav@gva.es](mailto:peman_jav@gva.es)

Telephone number: +34 622033355

**
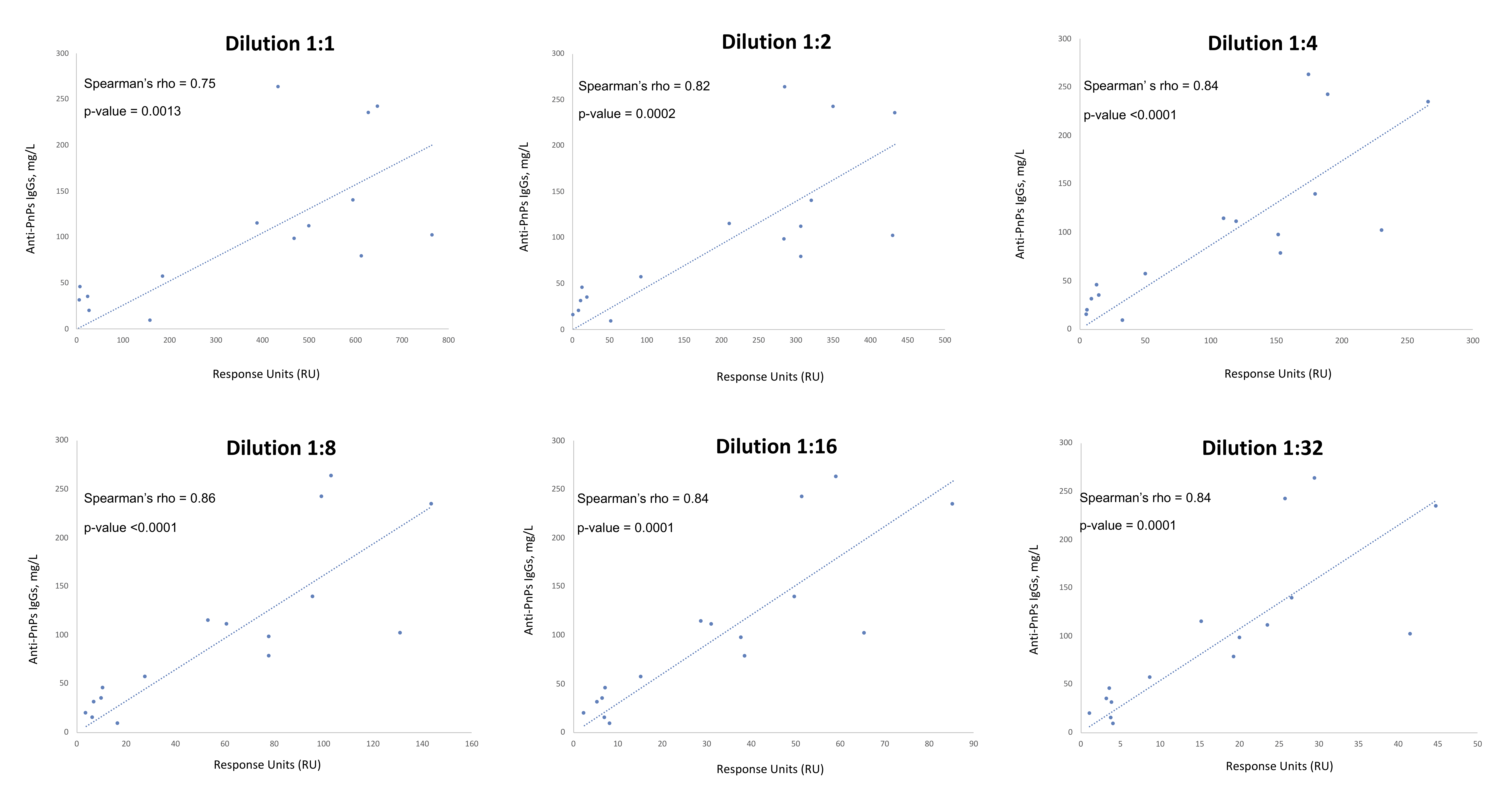
**

**Figure S1.** Spearman’s rank correlation analysis between SPR response (RU) and serum anti-PnPs IgGs levels determined by ELISA (mg/L) for all samples included in the study with a serum anti-PnPs IgGs concentration bellow 270mg/L, as determined by ELISA (SPR results for all the sample dilutions assayed in the study – 1:1, 1:2, 1:4, 1:8, 1:16, 1:32).

**
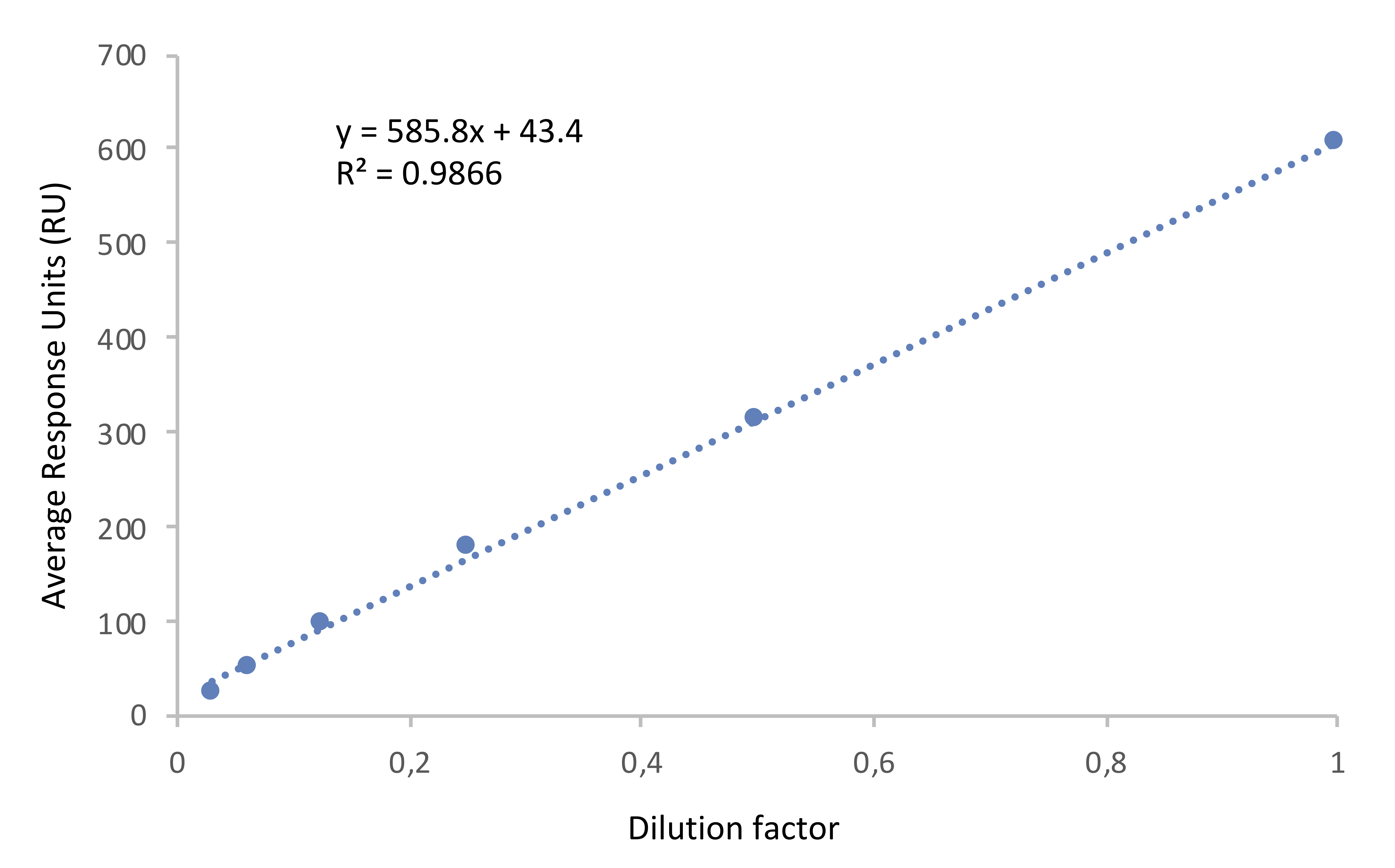
**

**Figure S2.** Linear correlation analysis between average SPR signal and sample dilution coefficient for all the serum samples included in the study.

**
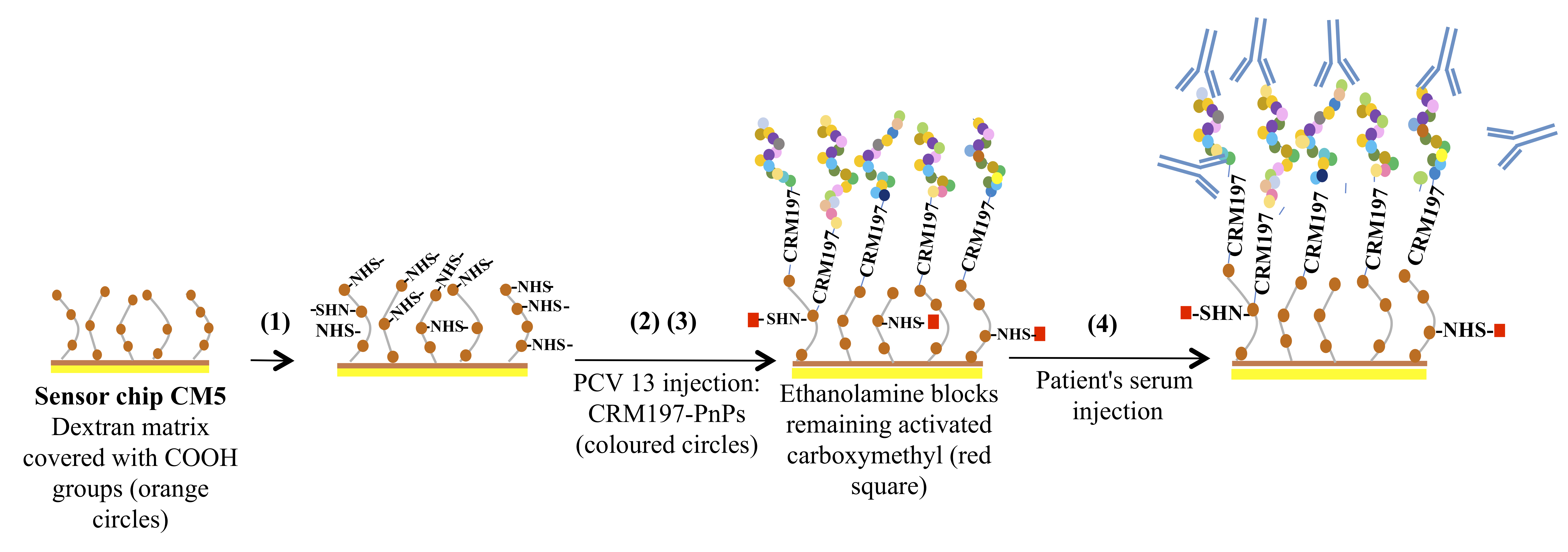
**

**Figure S3.** Scheme of the PCV13 immobilization using amine chemistry. The immobilization process consisted in three steps: (1) NHS/EDC were injected to activate the surface of the CM5 chip by modification of the carboxymethyl groups to N-Hydroxysuccinimide esters (-NHS-); (2) Diluted PCV13 was injected and the N- Hydroxysuccinimide esters spontaneously reacted with the amine groups in lysine residues of CRM197 to form covalent links between the PCV13 molecules and the CM5 chip surface; (3) Ethanolamine was injected to block free activated carboxymethyl groups and avoid non-specific binding in the SPR experiments; (4) Anti-PnPs IgGs generated by patients against the different epitopes present in the PCV13 are detected by SPR (anti-PnPs IgGs and CRM197).
